# Insights on the Role of Polyphenols in Combating Cancer Drug Resistance

**DOI:** 10.3390/biomedicines11061709

**Published:** 2023-06-14

**Authors:** Mohd Farhan

**Affiliations:** Department of Basic Sciences, Preparatory Year Deanship, King Faisal University, Al Ahsa 31982, Saudi Arabia; mfarhan@kfu.edu.sa

**Keywords:** cancer, drug resistance, antioxidants, plant polyphenols, pharmaceutical uses

## Abstract

Chemotherapy resistance is still a serious problem in the treatment of most cancers. Many cellular and molecular mechanisms contribute to both inherent and acquired drug resistance. They include the use of unaffected growth-signaling pathways, changes in the tumor microenvironment, and the active transport of medicines out of the cell. The antioxidant capacity of polyphenols and their potential to inhibit the activation of procarcinogens, cancer cell proliferation, metastasis, and angiogenesis, as well as to promote the inhibition or downregulation of active drug efflux transporters, have been linked to a reduced risk of cancer in epidemiological studies. Polyphenols also have the ability to alter immunological responses and inflammatory cascades, as well as trigger apoptosis in cancer cells. The discovery of the relationship between abnormal growth signaling and metabolic dysfunction in cancer cells highlights the importance of further investigating the effects of dietary polyphenols, including their ability to boost the efficacy of chemotherapy and avoid multidrug resistance (MDR). Here, it is summarized what is known regarding the effectiveness of natural polyphenolic compounds in counteracting the resistance that might develop to cancer drugs as a result of a variety of different mechanisms.

## 1. Introduction

The World Health Organization estimates that in 2020, around 10 million individuals lost their lives to cancer [1]. Cancers of the breast (2.26 million cases), lungs (2.21 million cases), colon/rectum (1.93 million cases), prostate (1.4 million cases), skin (non-melanoma) (1.2 million cases), and stomach (1.2 million cases) were the most prevalent types of cancer (1.09 million cases) [1]. It is worth noting that the cancer rate in the countries of the Middle East is rising at an alarming rate. It has been predicted that by the year 2030, the cancer rate will double [2,3]. Nonetheless, the rate of occurrence is significantly lower than in Western nations including the United States [3,4].

According to an age-standardized rate, Australia has the highest cancer rate in the world, with 452 new cases reported for every 100,000 people. Next comes New Zealand, with a rate of 422 per 100,000. The U.S.A. occupies a middle position in this list [4]. Yet, Middle Eastern countries report far lower numbers of cancer incidence. Egypt and Lebanon reported the highest rates, with 159 and 165 cases per 100,000 people, respectively [4]; Saudi Arabia and Sudan reported the lowest rates, with 96 and 95 cases per 100,000 people, respectively [3,4]

The prevalence of sedentary lifestyles (correlated with obesity, diabetes, and reduced physical activity) in Arab countries is considerable and is associated with an increased risk of cancer. The lack of exercise is likely due to a combination of factors, including cultural norms in some areas and the excessively hot weather that persists for many months of the year [5,6,7]. Having plenty of domestic help around the house can also play a role. Obese women, particularly post-menopausal women, have a relative risk of developing breast cancer that is more than twice as high as that of normal-weight women [8,9,10]. With the adoption of a Westernized lifestyle, smoking status and exposure to stress are projected to increase among Arabs. From 1990 to 2012, the estimated incidence of smoking was 12.5% higher than it had been in any GCC country, and this trend is projected to continue [11]. In addition, shisha is far worse than cigarettes since it contains significantly more nicotine [12]. For women who have smoked for more than ten years, the chance of developing breast cancer is roughly 10% higher than in never-smokers [7,13,14].

While cancer rates in Arab countries have risen somewhat in recent years, they are still significantly lower than those in the West. Fasting [15,16], food prepared according to specific recipes and rich with spices, vegetables, fruits, seeds, herbs, and olive oil; significantly lower rates of smoking and alcohol use, and genetic predisposition are all proposed explanations for the significantly reduced incidence of cancer in Arab countries [4,17,18,19,20,21,22,23,24,25].

Surgery is frequently used for the initial treatment of localized solid tumors. Nevertheless, targeted treatments, radiation, immunotherapy, and chemotherapy are used at later stages and/or after surgery [26]. Improvements in anticancer drugs have greatly enhanced patients’ quality of life and the length of time without relapse [27]. There are substantial limitations to the use of chemotherapeutic medicines in cancer treatment, including the medications’ solubility and instability, nonspecific drug distribution, and adverse effects related to systemic toxicity [28]. Although the initial therapy may be successful, some patients will experience a recurrence or relapse of their cancer. The failure of cancer treatment is now mostly attributable to acquired drug resistance [27]. Chemotherapy resistance in cancer cells can arise from either innate or acquired mechanisms [29]. The term “acquired drug resistance” describes a condition in which a patient develops immunity to a treatment that was successful in the past. Intrinsic chemoresistance occurs when there is something in the patient that makes the treatment ineffective from the start [30]. The heterogeneity of tumor cells is an underlying cause of resistance to chemotherapy. Heterogeneity is caused by self-renewing subpopulations of tumor cells, which have been identified as stem-like cancer cells. Several clones can be found within a single tumor, and all of them respond differently to chemotherapeutic drugs. Thus, a successful outcome may not be achieved by using a single drug to target tumor cells [31,32,33,34]. Cancer cells can become resistant to chemotherapeutic agents through a number of mechanisms, including upregulated drug efflux, altered drug target, apoptosis, and repair signaling pathways [35,36].

## 2. Therapeutic Potential of Polyphenols in Cancer

Polyphenols are a huge family of over 8000 plant compounds that share structural characteristics such as a three-membered flavan ring structure and numerous phenol units [36]. These natural substances (around 500 of them) can be found in a variety of foods, including fruits, green and black tea, coffee, red wine, chocolate, and seeds. Polyphenols are a group of organic compounds defined by the presence in them of numerous phenol structural units and found primarily in nature [36]. Subgroups of polyphenols can be established according to the number of phenol rings present and the structural factors that keep these rings together [36]. Phenolic acids, flavonoids, stilbenes, and lignans are the primary types of polyphenols. The concept of treating cancer patients with polyphenols is not novel. In the late 20th century, preliminary research on the anticancer effects of various polyphenols was conducted, and since then, our understanding of these advantageous agents has vastly improved [36,37]. These agents are highly advantageous and intriguing because they attack cancer cells in a variety of ways and target numerous cancer hallmarks. Initiation, promotion, and progression are the three major phases of cancer development [36,37]. The rapid and usual first stage in the development of cancer involves the ingestion of or exposure to a carcinogenic substance, which then interacts with chromatin and causes a mutation or epigenetic alteration [36,37].

The widespread severe side effects and subsequent toxicity generated by most conventional medicines have prompted cancer patients to turn their attention to natural products as a first line of defense [38,39,40]. Meanwhile, the pharmaceutical sector is researching and evaluating novel natural compounds for use in cancer therapy [41,42].

Despite attempts to promote cancer awareness, early detection, and new therapeutic treatments, progress in cancer therapy has been slowed by the emergence of drug resistance, the high costs of treatments, and the growing reports of secondary toxicity [43,44]. In addition, the expenses of treatment are inflated by the fact that most chemotherapeutic medications are known to cause unpleasant side effects such as nausea, vomiting, headache, musculoskeletal pain, anorexia, gastritis, oral ulcers, diarrhea, constipation, alopecia, neuropathy, and so on [45,46].

Natural supplements from different plants have been studied for their potential to treat cancer and its symptoms [47]. Here, natural products such as polyphenols may represent appropriate alternatives, especially when combined with other cancer medications to improve treatment efficacy and safety [48].

As stated earlier, resistance to chemotherapy in cancer cells can be intrinsic, resulting from pre-existing genetic abnormalities; such resistance typically becomes evident after the tumor has been exposed to anticancer medications (first leading to tumor regression) [49]. On the other hand, acquired resistance is created by the drug itself during treatment, leading to disease recurrence after initially successful chemotherapy [50,51]. Cancer cells can develop either intrinsic or acquired resistance when they undergo genomic or epigenomic alterations that promote signaling pathways other than the one targeted by the treatment in progress [52,53,54]. Hence, there are massive efforts to discover appropriate adjuvant drugs that aid in reversing the mechanisms of resistance, thereby favorably improving the efficacy of chemotherapies and, ultimately, disease remission in cancer patients.

## 3. Mechanisms of Cancer Chemoresistance

There are a number of hypotheses that have been put forward to describe the phenomenon of cancer chemoresistance as a reaction to both host-related factors that inhibit drug uptake across the plasma membrane and various genetic factors that lead to insensitivity to anticancer drugs. These hypotheses have been developed in response to both types of factors.

### 3.1. Cellular and Noncellular Mechanisms

**Figure 1:**
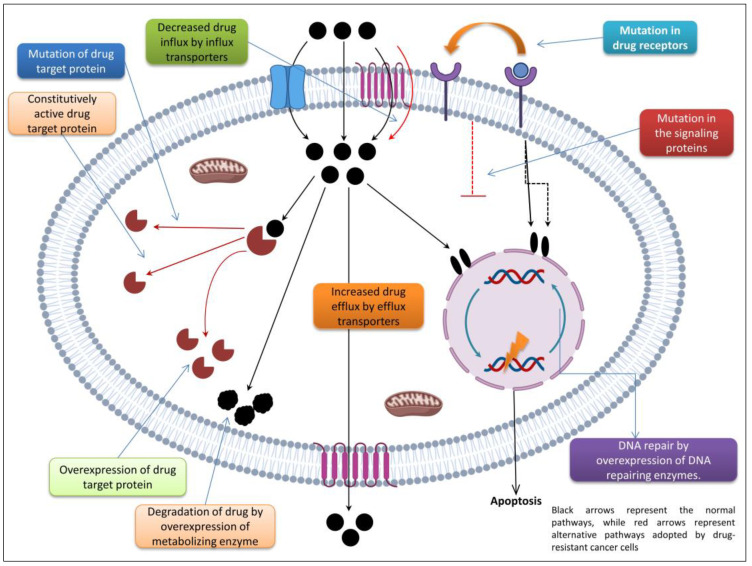
The several ways in which cancer cells develop resistance to chemotherapy.

Inherent or natural resistance to chemotherapy upon initial drug exposure is due to noncellular mechanisms (such as low drug bioavailability and metabolic inactivation), while cellular mechanisms involve a wide range of enzymatic, signal transduction, and cellular transport systems [55]. The mechanism depicted in Figure 1 is just one way by which cancer cells become resistant to chemotherapy. The oxygenation status and tumor vascularization appear to play a significant role in the difficulty of successful therapy in cancer patients, as tumor-associated vascular, structural, and functional abnormalities create an acidic environment for cells that are located distal to the vasculature, resulting in poor oxygenation and cell cycle arrest [56]. It has been found that noncycling cells are less sensitive to most chemotherapies [57] and that many malignancies that have hypoxia as an associated feature experience environmental insufficiencies that influence a variety of cellular pathways in the emergence of resistance [58].

Enzymatic or non-transport-based mechanisms can also mediate cellular MDR phenotypic activity by altering the biotransformation of anticancer drugs, which is important because metabolic activation is a preliminary step for many of these drugs to exert their deadly effects. In the case of cytarabine [59], for instance, it is first phosphorylated by deoxycytidine kinase to generate cytarabine monophosphate and then further phosphorylated to become cytarabine triphosphate, its active form. Thus, cancer cells acquire resistance by decreasing drug activation through the downregulation or mutation of enzymes implicated in this metabolic pathway, thereby evading the effects of such medications [59]. By overexpressing drug-metabolizing enzymes including CYP-4503A, glutathione-S-transferase (GST), and aldehyde dehydrogenase, cancer cells can become resistant to chemotherapy and other cytotoxic treatments [60].

### 3.2. Active Membrane Transport (ATP-Dependent Multidrug Transporters)

One of the most common mechanisms of resistance to many standard antineoplastics chemicals is the upregulation of membrane transporters, which are responsible for the efflux of cytotoxic compounds and the maintenance of intracellular drug concentrations below the threshold required for effective cytotoxicity [61]. These ATP-dependent multidrug transporters are part of the ubiquitous ATP-binding cassette (ABC) protein family [62] and help regulate the body’s uptake, distribution, and elimination of several pharmacologically active substances.

There are more than 48 genes that code for ABC transporters, and they fall into seven different gene families. P-glycoprotein (ABCB1, MDR1) is the most understood member of the MDR/TAP family, which also includes the homologs ABCB1-11, while the breast cancer resistance protein (BCRP, ABCG2) is the best-understood member of the ABCG family of transporters [63,64,65]. Different in gene location, amino acid sequence, structure, and substrate selectivity, these resistance-conferring proteins are all a part of the ABC superfamily [66,67,68]. Many of these have been defined in humans and are naturally expressed in a wide variety of healthy tissues as well as neoplasms [69]. P-gp is ubiquitous in tissues involved in the absorption, secretion, and transport of substrates across cellular plasma membranes, but its low expression makes it difficult to detect. MDR-ABC transporters are found in a wide variety of tissues [70]. The epithelial lining of the gut, endothelial cells, bone marrow progenitor xenocells, peripheral blood lymphocytes, and natural killer cells all contain ABCB1 in mammals, while adrenal cortex cells include ABCB2.

The “multiple drug-resistant” “MDR” phenotype is characterized by the ability of cancer cells to develop resistance to a wide variety of drugs, including those that share no structural similarity [71]. Many studies suggest that kidney, colon, pancreatic, and liver cancers, as well as malignancies originating from other tissues with naturally high levels of P-gp expression, may be inherently drug-resistant [72].

### 3.3. DNA Repair-Mediated Mechanisms of Resistance

Nucleotide excision repair, mismatch repair (MMR), double-strand break repair, base excision repair, and direct repair are all important DNA damage repair processes involved in conferring MDR to cancer cells. A better response of cancer cells to anticancer treatments (e.g., cisplatin and methotrexate) that kill cells via DNA damage-induced apoptosis is achieved through the downregulation of these mechanisms. For instance, in ovarian and breast cancers, selective cancer cell toxicity can be achieved by targeting the DNA repair-associated poly(ADP-ribose) polymerase (PARP) protein [73,74]. Despite the success of PARP inhibitors (PARPi), research in animals and humans has shown that resistance can develop to these drugs, even when they are given in combination [75,76]. The capacity of cancer cells to restore the DNA repair function by reversing a mutation in the BRCA gene is the primary mechanism of resistance to PARPi [77].

The ultimate aim of anticancer medications is to activate cell death or cell cycle arrest pathways. Thus, alterations to these pathways play a significant role in the emergence of MDR, perhaps through the avoidance of apoptotic pathways triggered by the acquisition of inactivating mutations in genes encoding apoptotic proteins (such as p53) or activating mutations in genes encoding antiapoptotic proteins [such as B-cell lymphoma 2 (Bcl-2)]. As mutant p53-associated MDR has been documented in over 50% of all malignancies, p53 role in drug resistance has been studied extensively [78,79].

### 3.4. Other Facilitators of the MDR Phenotype

The ubiquitin–proteasome system, which helps break down damaged proteins and controls development and the stress response, is another factor influencing the cancer MDR phenotype. This system’s overactivity is linked to cancer cell resistance, whereas its suppression leads to cell death and increases the sensitivity to chemotherapeutic drugs [80]. In addition, recent research has demonstrated that autophagy (i.e., cell “self-digestion”) plays a crucial role in chemoresistance by degrading chemotherapeutic drug molecules, which enables cancer cells to avoid drug-induced apoptosis [81,82]. Hence, blocking autophagy has been found to improve the effectiveness of chemotherapies [83,84]. Apoptosis-to-autophagy conversion has also been identified as a key mechanism of resistance to paclitaxel-induced cytotoxicity in breast cancer cells [85].

Cancer cell defense against oxidative stress and the generation of reactive oxygen species (ROS) is another factor contributing to the MDR phenotype. This defense involves the induction of the overexpression of various antioxidant enzymes by ROS-sensitive transcription factors (e.g., Nrf2), which permits cancer cells to overcome apoptotic signals and, thus, results in chemoresistance and tumor progression [86,87]. Finally, “oncogene addiction” [88] describes how many cancer cells become over-reliant on an oncogene, providing a rationale for developing targeted therapies aimed at oncogene-encoded proteins [89]. Nevertheless, medication resistance due to mutation of the targeted protein may compromise the treatments’ long-term efficacy. Mutations at “gatekeeper” residues, which prevent drugs from entering the kinase back pocket active site, are a common cause of resistance to tyrosine kinase (BCR/ABL and EGFR) inhibitors [90,91,92]. Hence, under therapeutic pressure, the most adaptive or resistant heterogeneous subpopulations of cancer cells will be selected. Following this, the tumor becomes dominated and repopulated by these clones, which renders it extremely resistant to the treatment [93].

## 4. Approaches to Overcome Drug Resistance

**Figure 2:**
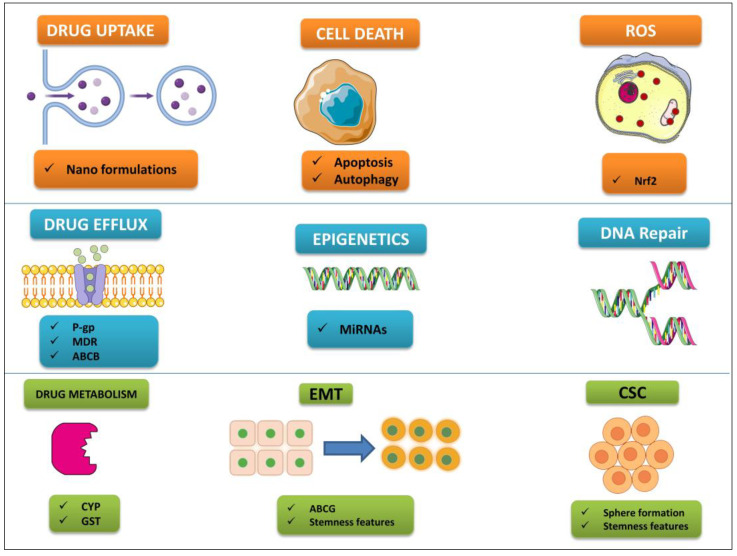
Mechanisms of cancer drug resistance and potential involvement of polyphenols.

Traditional cancer treatments typically provoke an unfriendly cellular environment, which can result in organelle dysfunction and the development of drug resistance in the area surrounding the tumor. Studies have begun to concentrate more on the use of bioactive anticancer chemicals due to their multi-target specificity, selectivity, and cyto-friendly nature [94,95]. This is done in an effort to mitigate the negative effects that are caused by conventional cancer treatments. In this context, the mechanisms of action of polyphenols have been investigated, in particular with respect to overcoming the drug resistance of certain cancers (see Figure 2).

### 4.1. Combination Therapy

Disease progression is connected with a variety of processes, including inherent and acquired medication resistance, both of which are heavily regulated by signal transduction pathways. Over the years, numerous strategies have been documented that successfully sidestep resistance pathways to cytotoxic, hormonal, and biologic drugs. As both genetic and epigenetic alterations are dynamic, they cooperate to keep the malignant phenotype in “homeostasis” in the face of a selection pressure [96]. Combination therapy, in which two or more chemotherapeutic agents with different mechanisms of action and different pathways to drug resistance are used, is widely accepted as a means of reducing the impact of drug resistance by preventing the use of alternative intracellular escape pathways necessary for tumor survival [97]. This strategy seeks to induce complementary/synergistic rather than merely additive effects; however, it usually comes with a high price tag, which makes it unattractive to many patients [98,99].

### 4.2. Pharmacological Inhibition of Membrane Efflux Transporters

The MDR phenotype has been studied extensively because it presents the greatest barrier to successful chemotherapeutic intervention against cancer. Several medications have been discovered to act as pharmacological antagonists of drug exporters, most notably P-gp. Depending on their affinity for a specific transporter(s) and their relative toxicity toward normal cells, MDR antagonists are categorized as either first-, second-, or third-generation drugs. These compounds function as modulators, reversal agents, or inhibitors of drug efflux [100].

Most modulators, however, have the issue of being carried by P-gp itself. Hence, their inhibitory impact is through competition, necessitating extremely high concentrations to be effective in vivo, leading to intolerable side effects such as cardiotoxicity and immunosuppression for the P-gp inhibitors verapamil and cyclosporine A, respectively [101]. Yet, there is another class of modulators that P-gp does not carry. To this end, vinblastine efflux is severely inhibited by hydrophobic steroids such as progesterone, megestrol acetate, and medroxyprogesterone, in contrast to what is observed for their more transportable hydrophilic counterparts [102]. While RU486 (an antiprogestin) is a powerful modulator in the lab, its hormone characteristics make it potentially dangerous in humans [103]. Clinical trials have been conducted using hydrophobic antiestrogens such as tamoxifen and its variants to treat breast cancer; however, because these compounds act as growth agonists in some uterine cells, they may increase the risk of endometrial cancer [104,105]. Inhibitors of growth factors and protein kinase C are two novel experimental techniques that have the potential to prevent or postpone the development of membrane transport-associated resistance [106].

### 4.3. Reversal of Drug Resistance by Naturally Occuring Polyphenolic Compounds

The systemic toxicity of chemotherapeutic drugs can now be minimized, while treatment efficacy is maximized, thanks to the development of successful alternative techniques. Dietary supplements and other phytotherapeutic substances are being studied for their potential to enhance the effectiveness of anticancer medications. Polyphenolic compounds with an extraordinary capacity to reverse drug resistance in vitro and in vivo may be discovered with the help of recent investigations in various drug-resistant cancer cell lines [107], and these compounds may be viable candidates for future clinical applications in cancer treatment [107]. The most recently investigated polyphenols and the chemoresistance mechanisms involved are summarized in Table 1 and Table 2.

As a result of the drawbacks of existing pharmacological MDR modulators, researchers are looking for novel molecules that may be effective at manageable levels and have fewer side effects. Hence, recent studies have revealed that natural compounds such as plant polyphenols may be effective MDR modulators (Table 1 and Table 2) [107]. Most of these natural chemicals are necessary for human survival, and flavonoids, in particular, have demonstrated exceptional profiles of safety and tolerability [36] even when administered at extremely high dosages (e.g., 1.0 g dietary polyphenol/day). Clinical experiments including these substances have shown promising findings, indicating that polyphenols may have an anticancer effect; furthermore, multiple preclinical research suggests that increasing polyphenol consumption or their usage as adjuvant therapy for cancer treatment is warranted [36].

### 4.4. Flavonoid Antagonism of Drug Efflux Transporters

Vegetables, fruits, herbs, and drinks include flavonoids, which are a class of polyphenolic compounds found in plants and characterized by their structural diversity due to the presence of two or more aromatic rings in their molecular makeup [36]. Flavones, isoflavones, flavonols, flavanones, chalcones, catechins (flavan-3-ols), and procyanidins are subclasses based on the presence or absence of certain substitutions [36,37]. The relevance of dietary flavonoids in cancer prevention or treatment has been extensively debated due to their wide range of bioactivities, including antioxidant, anti-inflammatory, antiviral, antiproliferative, and proapoptotic properties [36,37]. Flavonoids are well-known for their ability to interact with ABC transporters [129], impeding the binding of drugs to the transporters in either a competitive or an allosteric manner, therefore preventing the export of antineoplastics [130]. By binding to members of the ABC transporter family, flavonoids can either increase or decrease the transporters’ ATPase activity [131].

Flavonoids may potentially affect MDR transporter protein expression [132,133]. Many P-gp inhibitors have been identified and investigated as potential MDR-reversing drugs, meaning they can turn chemoresistance into chemosensitivity [134]. In particular, quercetin has been shown to have antiproliferative effects in a wide variety of cancer cell lines and animal models; hence, it is a well-established anticancer chemical. Despite the lack of clinical evaluation of its chemo-sensitizing impact, preclinical studies have shown significant promise of the use of this compound as an adjuvant to standard chemotherapy (Table 1 and Table 2), which may improve the therapeutic results for various malignancies, including melanomas. Finally, researchers [101] showed that quercetin might sensitize temozolomide-resistant DB-1 melanoma (wild-type p53) and SK-Mel-28 (mutant p53) cell lines through the regulation of p53 family members. Multidrug-resistant MCF-7 human breast cancer cells showed enhanced Adriamycin accumulation in the presence of quercetin, according to preliminary studies of this chemical as a P-gp modulator [101].

Certain flavonoids have the ability to reverse MDR, just like the well-known but highly lethal P-gp inhibitors verapamil and cyclosporine A [135]. By modulation of death receptor 3, a receptor for tumor necrosis factor (TNF), fisetin induces apoptosis, inhibits proliferation, and inhibits invasion in chemoresistant pancreatic cancer cells [101]. Several studies looked at the structure–activity connections of various flavonoids that were shown to impair P-gp-mediated transport. Flavanone (naringenin), flavone (baicalein), flavonols (flavanols), kaempferol, quercetin, myricetin, morin, fisetin, and two additional glycosides of quercetin were among the substances studied [101]. Drug buildup was observed in P-gp-overexpressing human epidermal carcinoma KB-C2 cells due to a suppression of the P-gp-mediated efflux of daunomycin, as demonstrated by the aforementioned studies. In terms of promotion of daunomycin accumulation, kaempferol was followed by quercetin, baicalein, myricetin, fisetin, and morin, among flavonoids [101]. Flavonols not only lowered P-gp expression but also impeded P-gp-mediated drug transport [101].

Flavonoids have been demonstrated to interact with the ATP-binding domains of P-gp and to reduce its ATPase activity, which is essential for drug translocation. P-gp has been well recognized as an ATP-driven drug export pump. Both the ATP-binding site and the steroid-interacting sections of P-gp cytosolic domain are targets for these drugs [136]. Certain flavonoids, in contrast to those that impede organic anion transport by binding to and activating MRP1, increase the transport of reduced glutathione (GSH) [137]. The largest (six-fold) increase in GSH transport by apigenin was observed at a dose of 30 mM, when multiple flavonoids (apigenin, naringenin, genistein, and quercetin) were employed to stimulate MRP1-mediated GSH transport. Flavonoids have been hypothesized to promote GSH transport by elevating MRP1 apparent affinity for GSH [138]. In addition, quercetin was shown to inhibit a heat shock factor in a P-gp-independent manner, reducing MDR [139]. The results of this study were corroborated by another that found flavonoid-rich foods caused tumor cells to accumulate more doxorubicin, allowing MDR to be reversed [140]. Research on this topic has yielded mixed results, although the vast bulk of the information points in a positive direction. Regarding this, various studies disproved the hypothesis that the isoflavone genistein has no effect on cancer cell P-gp-mediated MDR [141,142,143].

Research into the structural basis of MDR activity modulation by flavonoids and the establishment of a strong structure–activity relationship for the ultimate selection of a polyphenolic lead molecule have also been intensively pursued. P-gp active site has been used to test the binding affinities of several synthetic analogues [144]. It was also revealed [145] that several flavonoids may selectively revert BCRP-mediated drug resistance in various chemoresistant leukemia cell lines. The possible clinical benefits include enhanced efficacy and reduced toxicity of cancer chemotherapies made possible by these flavonoid BCRP inhibitors [145]. In addition to enhancing the bioavailability of doxorubicin, quercetin was demonstrated to decrease drug resistance by competitively inhibiting P-gp, MRP1, BRCP, and the metabolizing enzyme cytochrome P4503A4 [146]. As an added bonus, quercetin was found to exert antiproliferative and apoptosis-inducing actions on doxorubicin-resistant human gastric cancer cells [147]. 

As an ABC transporter, the BCRP protein plays a crucial role in healthy bodily processes [148]. Moreover, BCRP is crucial in regulating access to pharmaceuticals [149]. Currently, there are not many studies looking for particular inhibitors of this transporter. Several tamoxifen derivatives and the hormones estrone and estradiol have been found to overcome BCRP-mediated medication resistance in cancer [150]. There is also strong evidence that flavonoids can act as powerful BCRP-specific inhibitors. The accumulation of BCRP substrates is stimulated by silymarin, hesperetin, quercetin, and daidzein in resistant but not wild-type breast cancer cell lines [151]. The polyphenols genistein and naringenin were shown to interact with BCRP at higher concentrations than estrone [101]. Certain flavonols, such as kaempferide and kaempferol, and several flavones, such as acacetin, apigenin, chrysin, diosmetin, and luteolin, have great potential to reverse BCRP-mediated drug resistance, and they are safe enough to employed in clinical practice [101].

### 4.5. Dietary Polyphenol Inhibition of Oncogenic Signaling Pathways and Enhancement of Tumor-Suppressive Pathways

Dietary polyphenols have been found to act as chemopreventive agents by disrupting signal transduction pathways involved in carcinogenesis [152]. Cell cycle arrest, induction of apoptosis, antioxidant and anti-inflammatory actions, and suppression of angiogenesis are some of the outcomes of their interference with the cell’s natural processes [153]. A comparable effect of resveratrol on ovarian cancer cells’ sensitivity to cisplatin was observed through its ability to modulate cancer cell growth via MAPK inhibition [101]. There are many NF-kB-controlled genes implicated in tumorigenesis that have been shown to be downregulated when NF-kB activity is suppressed. These genes include tumor necrosis factor alpha (TNF-a), cyclooxygenase-2 (COX-2), cyclin D1 (cyclin D1), matrix metalloproteinase (MMP)-9 [154,155]. Curcumin and other polyphenols have inhibitory effects on NF-kB, which is important because most anticancer medicines activate NF-kB, leading to resistance [101].

Curcumin, like other phenolic chemopreventive agents, reduces the activity of the oncoprotein NF-kB and induces a proapoptotic cellular state by upregulating proapoptotic signaling proteins (p53 and p21) and downregulating cell survival proteins (phosphatidylinositol 3-kinase, protein kinase B, nuclear factor kappa B, and AP-1) [101].

Epigallocatechin gallate (EGCG), a component of green tea, inhibits the proliferation of human breast cancer cells by reducing the production of survivin, a member of the inhibitor of apoptosis protein family that is highly expressed in a wide variety of cancer cells and tissues [101].

### 4.6. Polyphenol Resensitization of Drug-Resistant Cancer Cells and Tumors

Milk thistle seed silibinin was proven in numerous in vitro and in vivo investigations to successfully sensitize cancer cells to apoptotic processes generated by a wide variety of chemotherapy drugs [156,157]. The invasiveness of human ovarian cancer cells resistant to paclitaxel was reduced in vitro after treatment with silibinin [158]. If this effect is replicated in vivo, silibinin would be a great contender as an adjuvant for paclitaxel, suggesting a potentially favorable chemotherapeutic regimen, particularly for patients with tumors that are resistant to paclitaxel alone. Cell growth inhibition by cisplatin or carboplatin in hormone-refractory DU145 prostate carcinoma cells was increased to 100% when silibinin was added, according to another study. This was due to a more robust G2-M arrest brought about by the reduced expression of Cdc2, cyclin B1, and Cdc25C, all of which are crucial for the transition from the G2 to the M phase [159]. Similarly, A549 lung cancer tumor xenografts were suppressed in growth, cell proliferation, and angiogenesis, and apoptosis was triggered when nude mice were orally fed silibinin (200 mg/kg) after being pretreated with doxorubicin (4 mg/kg). This anticancer impact was accompanied by less doxorubicin-induced systemic toxicity, which was a notable finding when the medicines were taken together [154]. As for methotrexate, silibinin dihemisuccinate increased the cytotoxic activity of methotrexate in a concentration-dependent manner, making methotrexate-resistant human rhabdomyosarcoma cell lines more sensitive to the drug [160].

Therapeutic approaches that target DNA repair pathways are an attractive option for making chemoresistant cancers more sensitive to treatment [161]. Inhibiting DNA repair and inducing death in MDR cells via modulation of autophagy were described following a short-term treatment with curcumin. Tumor angiogenesis and metastasis can be stifled by curcumin because it reduces the levels of vascular endothelial growth factor (VEGF) [162]. Downregulation of MMPs and VEGF is a possible mechanism by which quercetin, genistein, resveratrol, and other phenolic acids are able to suppress angiogenesis in in vitro cell-based systems and animal models [163,164]. The metastatic process may be slowed by the fact that polyphenolic chemicals also suppress the expression of vascular adhesion molecules [165,166].

Researchers found that the polyphenols in red raspberries can slow the expansion of a variety of cancer cell lines by inducing cell cycle arrest, apoptosis, or autophagy-associated cell death in a dose-dependent way [167]. At greater concentrations, berry extracts can also resensitize fulvestrant-resistant cells to treatment (1 mM). This suggests that berry extracts not only improve the response of resistant breast epithelial cells to the antiestrogen fulvestrant, but also raise the cell death response in susceptible cell lines [168].

### 4.7. Polyphenols and Cellular Metabolism in Cancer

Recent research suggests that regardless of their tissue of origin, nearly all cancer cells have a common trait of poor cellular energy due to uncontrolled metabolism [36,169,170,171]. There are significant differences between the metabolic features of cancer cells and those of normal cells when it comes to the generation of cellular energy [172]. Cancer cells, in contrast to normal cells, rely abnormally on anaerobic glycolysis and substrate-level phosphorylation to meet their energy demands, with glucose and/or glutamine as substrates [173]. Increased fatty acid production and elevated glutamine metabolism are just two examples of tumors’ unusual metabolic features [174,175]. Cancer cells, in contrast to normal cells, which typically rely on growth factor signaling to make use of energy resources [176], break down these resources via glycolysis and glutaminolysis to divert the intermediate products that are required for biosynthetic pathways and the maintenance of cellular growth and proliferation [177]. As a result of this metabolic transition, cells are no longer limited in their ability to grow and divide.

Damage to mitochondrial respiration, a possible cause of cancer, leads to fermentation (glycolysis) and dysregulated cellular metabolic processes [178]. In light of these hallmarks of cancer cells, the disease is increasingly thought of as an interchangeable process involving metabolic abnormalities and genetic mutability. Metabolic problems associated with genomic instability are commonly preceded by hypoxia and mitochondrial malfunction, which have been linked to cancer and resistance [179,180]. If left untreated, this issue can progress to the point of somatic genomic instability, which in turn can cause additional mitochondrial abnormalities and metabolic rigidity in tumor cells [181,182]. Recent studies have found that energy restriction mediated by interfering with glucose or glutamine metabolic pathways is closely related to improvement in cancer therapeutic response and tumor regression [183,184,185], lending credence to the hypothesis that dysregulated cellular metabolism is linked to drug resistance in cancer therapy. Therefore, compounds that affect particular metabolic components and enzymes (such as glucose transporters, hexokinase, pyruvate kinase M2, lactate dehydrogenase A, glutaminase, and fatty acid synthase) associated with dysregulated cellular glycolysis, glutaminolysis, and fatty acid synthesis may improve the therapeutic response or decrease resistance to conventional anticancer agents [186]. Combining chemotherapeutics with inhibitors of cellular metabolism is also regarded to be promising; however, this approach to overcoming chemoresistance has not been thoroughly investigated [187].

The majority of bioactive chemicals with medicinal potential are found in nature. Flavonoids and dietary polyphenolic chemicals are just two examples of these food components that have been shown to have anticancer action [36,180]. Dietary polyphenols have been linked in numerous studies to controlling glucose and lipid metabolism via a variety of mechanisms and pathways [188]. In several types of cancer cells, polyphenols were shown to regulate glucose transporter activity. Well-known inhibitors of glucose absorption include myricetin, quercetin, genistein, cyanidin, hesperetin, naringenin, and catechin [189,190,191]. To destroy cancer cells and make them more sensitive to chemotherapy, polyphenols are used to inhibit their basal glucose transport [192]. EGCG, curcumin, and resveratrol are three of the most researched and potentially cancer-preventing polyphenols. These compounds’ chemopreventive effects can be explained in a number of different ways, including the fact that they interfere with or even reverse the carcinogenic process by influencing molecules in the intracellular signaling network that play a role in the development and/or maintenance of cancer [193,194,195].

In addition to mediating hypoxia-induced MDR in cancer cells by preventing drug-induced apoptosis and lowering intracellular drug accumulation, polyphenols can inhibit the activation of hypoxia-inducible factor-1 (HIF-1a), the central molecule responsible for controlling the expression of glucose transporters and the other key glycolytic enzymes [196,197].

By blocking the production of HIF-1 and its target genes GLUT-1, hexokinase II (HKII), and VEGF, polyphenols are thought to sensitize cancer cells to chemotherapy [198]. Additionally, many polyphenols can inhibit the mitochondrial metabolism, a key mechanism in cancer development [199,200]. Reduced mitochondrial membrane potential and an imbalance in the cellular energy molecule (ATP/ADP) as a result of ATP synthase inhibition are two telltale signs of mitochondrial malfunction. Alterations in the expression of apoptotic markers such as Bax and Bcl-2 are also linked to mitochondrial malfunction [201]. Polyphenols found in tea and curcumin have been shown to trigger apoptosis in a variety of cancer cell types by disrupting mitochondrial metabolism and function [202,203,204]. Several studies point to mitochondrial dysfunction in cancer as a result of redox changes. Examples include a new mechanism for the regulatory role of curcumin in this phenomenon [205]. Last but not least, one intriguing approach to inhibiting cancer cell survival and resistance is to interfere with their lipid metabolism. Many natural polyphenols’ anticancer effects are thought to stem from their capacity to regulate the lipid metabolism [206], specifically by blocking the overexpression and activation of the enzyme fatty acid synthase [207].

### 4.8. Anti-Metastatic and Epigenetic Effects Exerted by Polyphenols

The term “metastasis” refers to the process by which cancer cells spread from their original location to other parts of the body, where they might cause even more damage. Microenvironmental components, such as stromal fibroblasts and immune cells, influence tumor cell behavior and promote metastasis. The key processes that prime tumor cells for infiltration are cellular motility, hypoxia, epithelial–mesenchymal transition (EMT), and angiogenesis [208,209,210]. Studies have shown that matrix metalloproteinases (MMPs), transforming growth factor beta (TGF-β), and TP53 all play critical roles in metastasis management [208,209,210].

Multiple stages of this process have been demonstrated to be susceptible to modification by polyphenols. For example, curcumin reduces the metastatic properties of cancer cells by influencing EMT-related proteins such as vimentin, fibronectin, β-catenin, and E-cadherin as well as genes expressed in cancer stem cells such as Oct4, Nanog, and Sox2 [211]. Additionally, quercetin and its derivatives are able to block EMT, MMP secretion, NF-kB, and cancer cell migration and metastasis [212,213,214,215]. Resveratrol inhibits metastasis by reversing EMT via AKT/GSK-3β/Snail signaling and reducing MMP-2 and 9 as well as Smad2 and 3 levels [216].

Tumor development, metastasis, and therapeutic resistance stem from epigenetic dysregulations and aberrations [217]. Cancer is characterized by epigenetic abnormalities including, but not limited to, DNA methylation, histone modifications, chromatin/nucleosome remodeling, and miRNA regulation [217].

Among the polyphenols, curcumin is one of the most effective at preventing these modifications from helping cancer cells. The enzymes known as histone deacetylases (HDACs) remove acetyl groups from histones, thereby contributing to gene silencing [218]. Curcumin has been shown to inhibit these enzymes and hence control the growth and death of cancer cells [219]. Curcumin has been shown to block the activity of histone deacetylases 1 (HDAC1), 2 (HDAC2), 3 (HDAC3), 4 (HDAC4), and 8 (HDAC8) [219,220,221]. Histone acetyltransferases (HATs) are another group of enzymes that can be used to foretell whether or not cancer cells will proliferate and survive. Some studies have found that curcumin inhibits these enzymes, and one of them is p300 [222,223]. This inhibition might occur directly or indirectly.

In addition, curcumin inhibits DNA methylation in the promoter region of numerous cancer-related genes by lowering the amount of DNA methyltransferase 1 (DNMT1) [224,225]. This includes the tumor suppressor gene Wnt inhibitory factor-1 (WIF-1) [226], FANCF [227], Nrf2 [228], Neurog1 [229], and RARβ2 [230].

Curcumin regulates microRNAs (miRNAs) including miR-125-5p, miR-19a, miR-9, and miR-145 in nasopharyngeal, breast, and ovarian cancer and leukemia [231]. Comparatively, resveratrol can affect the expression of miR-200, miR-122-5p, miR-20, and miR-633, just like other polyphenols can [232,233,234,235]. In cancer cells, quercetin controls the expression of many microRNAs [236,237,238,239], including miR-16, miR-22, miR-200b-3p, and miR-146a. In addition to EGCG, genistein regulates a number of other microRNAs [240,241] that play a role in correcting epigenetic changes in cancer cells.

### 4.9. Targeting Cancer Stem Cells Using Polyphenols

Less than one percent of the cells in a tumor are cancer stem cells (CSCs), a subtype of tumorigenic cells that are distinguished by their capacity for self-renewal and multipotency [242,243]. Researchers found that CSCs are to blame for the poor response of tumors to chemotherapy and radiation in virtually all cases [244,245,246,247,248,249,250,251,252]. The main reasons why CSCs provide resistance to our common therapies are their stemness features and the fact that they slow down the cell cycle, possess an anti-apoptotic machinery, have a high capacity for repairing DNA damage, and have the power to establish a proper environment for cancer growth [242,243,253,254]. Other factors that aid CSCs in this process include the Notch, Wnt, STAT3, PI3K/Akt, and NF-kB signaling pathways, protective autophagy, metabolic flexibility, and oxidative modulators [254,255]. It may be said that CSC targeting is a viable strategy for lowering tumor resistance and raising the efficacy of our standard medicines.

Recently, a novel idea for treating resistant tumors has emerged: using polyphenols to specifically target these cells. Curcumin has been shown to have anti-CSC properties in a variety of cancer types, including colon [256], pancreatic [257], liver [258], breast [259], and brain [260]. Colon cancer cells treated with curcumin had reduced levels of CSC markers such as CD44, CD133, and CD24 and a diminished ability to form a sphere [256]. Curcumin, either alone or in combination with irinotecan (CPT-11), induced apoptosis in CSCs, leading to less resistance to a chemotherapeutic medication [256]. Curcumin was also linked to suppressing CSC stemness by blocking the EZH2 polycomb repressive complex 2 (PRC2) subunit [257,261]. Curcumin increased the sensitivity to gemcitabine in pancreatic cancer cells by inhibiting a long non-coding RNA called PVT1 [257]. Curcumin can also influence genes including Nanog, Sox2, and Oct4 that are involved in stemness [259]. Curcumin’s capacity to reduce antiapoptotic protein levels and raise proapoptotic protein levels in CSCs is another method by which it reduces chemoresistance [262]. Bcl-2 and Bcl-w are examples of the former, while Bax, Bak, Bad, Bik, and Bim are examples of the latter. By doing so, mitomycin C resistance in breast cancer can be lowered [262]. Curcumin has shown promise in the treatment of brain cancer when used in conjunction with nanomedicine [260]. Anti-aldehyde dehydrogenase-grafted curcumin-loaded nanoparticles not only improved curcumin’ capacity to cross the blood–brain barrier but also released the polyphenol steadily over time [260]. Curcumin’s ability to make breast cancer cells more vulnerable to mitomycin C was also shown in an in vivo investigation [263]. Breast cancer stem cells were inhibited by a combination of curcumin and paclitaxel [263]. This action was mediated through the ATP-binding cassette (ABC) transporters ABCG2 and ABCC1.

The polyphenol EGCG can also inhibit CSC features including proliferation and migration in nasopharyngeal cancer cells [264]. In an osteosarcoma cell line, baicalin, a flavone, was tested for its role in EMT and was found to diminish anoikis-related resistance by inhibiting the EMT-inducing transcription factors Snail1 and Slug [265]. In addition to reversing EMT and decreasing the cisplatin resistance of lung cancer cells (in vivo and in vitro), baicalin also suppressed the PI3K/Akt/NF-kB pathway [266].

The anti-resistance effects of EGCG in cancer stem cells were further supported by an in vivo study. Researchers found that by inhibiting colorectal cancer stem cells with EGCG, they were able to increase the levels of tumor-suppressing microRNAs and make the cells more sensitive to the chemotherapy drug 5-fluorouracil (5-FU) [267].

### 4.10. Clinical Studies of Polyphenols with Promising Anticancer Effects

Polyphenols have emerged as a promising new class of therapeutic agents, and their usage in clinical studies to investigate potential health benefits in various cancers has increased dramatically in recent years [268]. Curcumin, EGCG, resveratrol, quercetin, apigenin, and kaempferol were all shown to have strong anticancer effects, but most of these research was conducted in preclinical models [269]. Experimental results in cellular/animal models cannot be considered to be applicable to people, mainly because of variations in genetics and metabolism; hence the bioactivities of polyphenols must also be explored in detail in humans. Most of these investigations will focus on learning more about the substances’ pharmacokinetics, pharmacodynamics, and safety and the processes by which they manifest their effects. Table 3 summarizes the results of some recent clinical trials involving a variety of polyphenols for the treatment of various cancers.

Moreover, polyphenols, when given as an adjuvant, were demonstrated to increase chemosensitivity in tumor cells, as reported in Table 4.

Polyphenols have the potential to be employed either alone or in combination with other cancer therapies. Exploring the mechanisms of action of these compounds could lead to greater therapeutic success in the treatment of cancer [268,269]. Prior to their usage as prescription medications, however, the safety of these substances must be established by additional research, which may include human subjects and various pharmacokinetic parameters. Moreover, more clinical trials could investigate the development of a standardized extract or dosage to be used as a successful cancer treatment regime.

## 5. Polyphenol Bioavailability and Metabolism

Recent research on the bioavailability of polyphenols has shown that even when consumed in relatively large concentrations, these compounds have a low bioavailability. Their poor bioavailability is a major roadblock to their application in pharmacology because of the molecular structure changes that occur during digestion, absorption, and distribution as a result of interactions with food, digestive enzymes, and transporters in the intestine and with blood proteins [270].

It is considered that polyphenols are bioavailable and can efficiently reach the target tissue. Knowing how they are ingested, processed, and eliminated from the body is crucial. The chemical complexity of polyphenol-rich diet, as well as other characteristics including the degree of polyphenol polymerization and conjugation with other compounds and phenols, makes absorption studies difficult to conduct [270]. Most polyphenols in food are not digestible because they are bound to esters, glycosides, or polymers. As the body treats polyphenols as foreign invaders after they have been ingested, their bioavailability is limited compared to that of micro- and macronutrients [270,271].

Similar to metabolic detoxification, polyphenol metabolism can lessen their cytotoxicity by increasing their hydrophilicity and making them easier to eliminate via the urinary or the biliary system [270]. The rate and degree of absorption and the kind of circulating metabolites in the plasma are determined by the structure of polyphenols, not by their content. These chemicals are either easily absorbed in the small intestine (monomeric and dimeric polyphenols) or reach the large intestine almost unchanged (oligomeric and polymeric polyphenols) [270,271].

Only about 5–10% of polyphenols that are consumed are absorbed in the small intestine, according to some literature. Less complex polyphenolic chemicals are able to undergo hydrolysis and biotransformation in enterocytes and subsequently in hepatocytes following absorption. This process leads to a cascade of hydrophilic conjugated metabolites (methyl, glucuronide, and sulfate derivatives) that are rapidly absorbed in the bloodstream and either transported to various organs or eliminated in the urine [270]. Because each phenolic molecule may give rise to ten other compounds throughout metabolism [270], it is challenging to trace the fate of each component in the body. Metabolic activities typically transform phenolic antioxidants into entirely new compounds, making the parent phenol nearly impossible to identify [270]. Phase I metabolism occurs in enterocytes when xenobiotics are oxidized, reduced, or hydrolyzed to introduce or expose a functional group, such as a hydroxyl group, particularly for conjugation (phase II metabolism) [270,271]. However, the hydroxyl groups on the phenolic aromatic ring are successfully conjugated to glucuronide, sulfate, and/or methylated metabolites through the action of uridine-5-diphosphate glucuronosyltransferase (UGT), sulfotransferase (SULT), and catechol-O-methyltransferase (COMT) [270,271]. The meta position is the preferred conjugation site for catechol antioxidants [270]. Para-position conjugation with a glucuronic acid or sulfate group is less common than meta-position conjugation with a methyl group, although it does happen [270,271,272].

The poor knowledge available regarding the absorption of polyphenols in humans is a major hurdle that needs to be addressed to increase the efficacy of these compounds as chemopreventive and chemotherapeutic agents for the benefit of health. Nanoparticle encapsulation is a possibility that may allow for more precise targeting and controllable release. However, further research is required to put this idea into general practice for polyphenol-based cancer therapies.

## 6. Conclusions and Future Perspectives

New approaches are being considered to improve the therapeutic outcomes as the incidence of cancer rises and standard treatments lose their efficacy. There has been a rise in interest in alternative medicines because of the negative outcomes that can result from conventional treatments. Scientists advocate for the use of polyphenolic chemicals found in nature, which can spare healthy tissues while killing cancers. Polyphenols such as curcumin, quercetin, EGCG, resveratrol, and apigenin have shown efficacy as potential cancer therapy agents, especially when paired with chemotherapeutic drugs such as cisplatin, 5-fluorouracil, docetaxel, paclitaxel, gefitinib, and others to induce synergistic effects. They are highly effective as antioxidants and also reduce inflammation, slow cell proliferation, inhibit angiogenesis, inhibit metastasis, and promote apoptosis. They were shown to perform exceptionally well in tests involving several different kinds of cancer, including those of the lung, breast, liver, colon, and stomach. They were able to trigger apoptosis and block the development and division of numerous cell lines. They can also be preloaded into certain biomaterials to provide a steady release and a simultaneous therapeutic and regenerative effect. In order to fully understand the mechanism involved in the polyphenol-induced regulation of cancer, additional research is needed, particularly on in vivo models and in additional clinical trials. The immunomodulatory impact of polyphenols, which can boost the efficacy of treatments such as immune checkpoint blockade, also needs a thorough evaluation. The potential for polyphenol treatment to reduce toxicity and adverse effects has contributed to its increased prominence in cancer therapy. However, this technique must be used with caution, as it can lead to the development of dormant tumor cells, which can cause the disease to recur.

## Figures and Tables

**Table 1:** Summary of polyphenol and chemotherapeutic combination therapies in in vitro preclinical investigations.

Polyphenol	Cancer	Chemotherapy Drug	Dosage	Assay	Molecular Effect	Conclusion	Reference
Curcumin	Lung cancer	Cisplatin	2–32 µM curcumin + 0.5–8 µg/mL cisplatin.	A549, H1299, NCI-H460 cell lines	Upregulating the levels of CTR1 and Sp1 to increase more Pt^2+^ uptake.	Enhancing sensitivity and antitumor effects of CIS in NSCLC	[108]
	Colorectal cancer	Oxaliplatin	HCT116 and SW480 cells 0–8 µM curcumin + 0.5–32 µM oxaliplatin; HCT116/oxaliplatin cells 4 µM curcumin + 8 µM oxaliplatin	HCT116, SW480, HCT116/Oxaliplatin drug-resistant cell lines	Inhibition of TGF-β/Smad2/Smad3 signaling	Inhibition of cell proliferation and reduced tumor weight and volume	[109]
	Breast cancer	Doxorubicin	25 µM curcumin + 5 µM doxorubicin	MCF-7/doxorubicin drug-resistant cell line	Reduced Aurora-A expression. Triggered p53 stabilization. Growth arrest and apoptosis induction	Reversed doxorubicin insensitivity and increased sensitivity in doxorubicin-resistant MCF-7 and MCF-7 cell lines	[110]
Quercetin	Liver cancer	Doxorubicin; 5-fluorouracil (5-FU)	40–160 µM quercetin + 0.2–125 µg/mL doxorubicin/5-FU	BEL-7402 and BEL-7402/5-FU drug-resistant cell lines	Inhibition of FZD7/β-catenin pathway and ABCB1, ABCC1, and ABCC2 efflux pump	Enhanced doxorubicin and 5-FU sensitivity	[111]
	Colorectal cancer	Doxorubicin	33 µM quercetin + 0.5 µM doxorubicin	SW620/ doxorubicin drug-resistant cell line and SW620/Ad300 cell line	Reversed P-gp-mediated drug resistance, increased intracellular doxorubicin accumulation; modulated glutamine metabolism in doxorubicin-resistant cells by inhibition of SLC1A5	Reversed MDR, enhanced sensitivity to doxorubicin	[112]
Resveratrol	Lung cancer	Gemcitabine	10 µM RES + 1 µM gemcitabine	HCC827 cell lines and HCC827	Downregulation of mRNA and protein levels of ENG, activation of ERK signaling pathway	RES promoted tumor microvessel growth, increased blood perfusion and drug delivery into tumor that resulted in enhanced anticancer effect of GEM	[113]
	Gastric cancer	Cisplatin	20 μM RES + 1 μg/mL cisplatin	AGS cell line	Upregulation of Bax and the cleaved form of PARP, downregulation of Bcl-2, increased PERK, p-eIF2α, and CHOP protein levels. Activation of PERK/eIF2α/ATF4/CHOP signaling pathway, induction of G2/M cell cycle arrest	Synergistically inhibited cell growth of cancer cell lines	[114]
EGCG	Breast cancer	Arsenic trioxide and/or irradiation	10–100 µM EGCG + 2 Gy radiation; 10–100 µM EGCG and 4 µM arsenic trioxide. 10–100 µM EGCG, 4 µM arsenic trioxide and 2 Gy radiation.	MCF-7 cell lines	Bax upregulation and Bcl-2 downregulation	Combination of EGCG and Arsenic trioxide with or without radiation showed synergistic effects in breast cancer treatment visible in the rise of cell death	[115]
	Lung cancer	Doxorubicin	0.5 μM EGCG + 0–100 μM doxorubicin	Nonresponsive A549 cell line	Decreased drug efflux, MDR signaling, and invasiveness. Increased drug internalization, cell cycle arrest, stress induced damage, and cell death	EGCG reversed the compromised functionality of doxorubicin in a nonresponsive A549 cell line and improved its oxidative damage-mediated antitumor effect by modulating redox signaling	[116]
Apigenin	Colorectal cancer	5-FU	20 µM apigenin + 20 µM 5-FU	HCT116 and HT29 cell lines	Inhibited the upregulation of TS induced by 5-FU. Increased reactive oxygen species production, intracellular and intramitochondrial Ca^2+^ concentrations, and mitochondrial membrane potential	Apigenin enhanced the efficacy of 5-FU by potentiating HCT116 cell apoptosis and enhancing cell cycle disruption. Acquired resistance to 5-FU was reduced	[117]
	Breast cancer	Cisplatin	5–100 μg/mL apigenin + 5–100 μg/mL cisplatin	MDA-MB-231 and HCC1806 cell lines	Inhibition of telomerase activity. Down-regulation of hTERT, Hsp90, and p23 at transcriptional and translational levels	Apigenin and cisplatin synergistically inhibited telomerase activities by reducing the catalytic subunit of the enzyme	[118]

**Table 2:** Summary of polyphenol and chemotherapeutic combination therapies in in vivo preclinical investigations.

Polyphenol	Cancer	Chemotherapy Drug	Dosage	Assay	Molecular Effect	Conclusion	Reference
Apigenin	Liver cancer	Paclitaxel	1 mg/kg/day apigenin + 3.5 mg/kg/day paclitaxel	Balb/c nude mice	Suppressing the intratumoral expression of HIF-1a via inhibiting the AKT/p-AKT pathway and the expression of HSP90 simultaneously	Apigenin reduced hypoxia-induced paclitaxel resistance in hypoxic tumors	[119]
	Lung cancer	Navitoclax	25 mg/kg apigenin + 100 mg/kg ABT-263	BALB/c nude mice	Upregulated the expression of Noxa by targeting the AKT–FoxO3a pathway and inhibited ERK	Apigenin synergized with ABT-263 by suppressing the growth and proliferation of tumor cells in vitro and in vivo	[120]
EGCG	Lung cancer	Cisplatin	EGCG (1.5 mg/mouse/day IP) for 5 days and cisplatin (2 or 4 mg/kg IP) on day 5; EGCG (1.5 mg/mouse/day) and single-dose cisplatin (2 mg/mouse) on day 0 or 5	A549 cell xenograft bearing BALB/c nude mice	Increased cisplatin concentration in tumor tissue and tumor growth delay due to EGCG-induced vascular normalization	EGCG synergistically potentiated cisplatin antitumor efficacy especially when cisplatin was applied during the vascular normalization window	[121]
	Liver cancer	Sorafenib	100 mg/kg EGCG + 10 mg/kg sorafenib	Diethyl nitrosamine-induced hepatocellular carcinoma in Wistar albino rats	Histopathological observations revealed a satisfying decline in tissue degeneration and hyperchromatism. Significantly lower alpha-fetoprotein and liver enzyme levels were detected, as well as a greater antioxidant capacity	EGCG and sorafenib combination had a comparable effect as that of standard-dose sorafenib. The combination resulted in enhanced chemoprotection and is considered effective against hepatocellular carcinoma	[122]
Resveratrol	Colorectal cancer	5-FU	10 mg/kg b.w. resveratrol p.o./day + 12.5 mg/kg b.w. 5-FU i.p. injected on days 1, 3, and 5; repeated every 4 weeks for 4 months	Methyl nitrosourea-induced colon cancer in male albino rats	Decrease of NF-κB and reduction of COX-2, induced p53 gene expression	Resveratrol biochemically modulated and enhanced the therapeutic effects of 5-FU	[123]
	Lung cancer	Gemcitabine	25 mg/kg gemcitabine i.p. 2×/week + 1 µmol/kg resveratrol 5×/week	HCC827 xenografts in nude mice	Downregulation of mRNA and protein levels of ENG, activation of ERK signaling pathway	Resveratrol promoted tumor microvessel growth, increased blood perfusion and drug delivery into tumor that resulted in enhanced anticancer effect of gemcitabine	[124]
Quercetin	Breast cancer	Cisplatin	30 mg/kg quercetin + 7 mg/kg cisplatin	Breast tumor-bearing mouse model	Inhibited tumor growth and reduced renal toxicity	Synergistic effect; inhibited renal toxicity induced by cisplatin	[125]
	Liver cancer	Sorafenib	7.5 mg/kg/day sorafenib, 2 h later 50 mg/kg/day quercetin	Chemically induced HCC rat model	Suppressed proliferation, enhanced apoptosis and necrosis	Synergistically increased anticancer effect and increased liver recovery	[126]
Curcumin	Lung cancer	Gefitinib	1 g/kg CUR + 100 mg/kg gefitinib	BALBL/c mice	Inhibition of Sp1/EGFR activity to induce autophagy-mediated apoptosis	Reduction in tumor volume. Elevated the sensitivity to gefitinib in NSCLC patients with mutated EGFR	[127]
	Liver cancer	5-FU	56.65 mg/kg curcumin + 10 mg/kg 5-FU	BALB/c nude mice	Decreased expression of NF-κB protein in the nucleus. Increased expression of NF-κB protein in the cytoplasm. Downregulation of COX-2 expression	Synergistic effects and in vivo tumor growth inhibition	[128]

**Table 3:** Recent clinical trials using polyphenols against cancers (adapted from ref. [268]).

Polyphenol	Type of Cancer	Outcome	Status
Resveratrol	Liver Cancer	Improvement of the Metabolic Profile of Liver Cells	Withdrawn
Caffeic Acid	Esophagus Cancer	1-Year Overall Survival (OS)	Ongoing
Quercetin	Primary Prevention of Prostate Cancer	Log2-Transformed PSA Measurements	Ongoing
Retinol	Lung Cancer	Prevention Of Lung Cancer	Completed
Alpha-Tocopherol	Head and Neck Neoplasms	Prevention of Second Primary Cancers	Completed
Grapes	Colon Cancer	Localization of β-Catenin and Wnt Target Gene Expression in Intestinal Mucosa	Withdrawn
Retinol	Lung Cancer	Lung Cancer Incidence	Completed

**Table 4:** Polyphenols that make tumor cells more sensitive to chemotherapeutic drugs (adapted from ref. [107]).

Polyphenol	Chemotherapy Drug	Result
Apigenin	Cisplatin	Inhibits growth of drug-resistant colon cancer cells while inducing autophagy
Resveratrol	Cisplatin	Induces autophagic and apoptotic death in drug-resistant oral cancer cells
Curcumin	5-Fluorouracil	Exerts synergistic effects with the chemotherapeutic drug by impairing AMPK/ULK1-dependent autophagy
EGCG	Cisplatin	Increases sensitivity of CAR cells, apoptosis, and autophagy by AKT/STAT3 pathway
Curcumin	Docetaxel	Leads to induction of apoptosis and autophagy through PI3K/AKT/mTOR pathway
Resveratrol	Gefitinib	Overcomes drug resistance while inducing apoptosis, autophagy, and senescence in PC9/G NSCLC cells
Curcumin	Gefitinib	Enhances the efficacy of the drug and overcomes EGFR-TKI resistance in NSCLC patients with wild-type EGFR and/or KRAS mutation

## Data Availability

Not applicable.
